# Treatment response assessment with (*R*)-[^11^CPAQ PET in the MMTV-PyMT mouse model of breast cancer

**DOI:** 10.1186/s13550-018-0380-x

**Published:** 2018-04-03

**Authors:** T. Tegnebratt, L. Lu, S. Eksborg, A. Chireh, P. Damberg, S. Nikkhou-Aski, T. Foukakis, H. Rundqvist, S. Holmin, R. V. Kuiper, E. Samen

**Affiliations:** 1 Department of Clinical Neuroscience, Karolinska Institutet, SE-17176 Stockholm, Sweden; 2 Department of Oncology-Pathology, Karolinska Institutet, SE-17176 Stockholm, Sweden; 3 Department of Cell and Molecular Biology, Karolinska Institutet, SE-17176 Stockholm, Sweden; 4 Department of Women’s and Children’s Health, Karolinska Institutet, SE-17176 Stockholm, Sweden; 5 Department of Neuroradiology, Karolinska Experimental Research and Imaging Center, Karolinska University Hospital, SE-17176 Stockholm, Sweden; 6 Department of Comparative Medicine, Karolinska Experimental Research and Imaging Center, Karolinska University Hospital, SE-17176 Stockholm, Sweden; 7 Core Facility for Morphologic Phenotype Analysis, Laboratory Medicine, Karolinska Institutet, SE-14183 Huddinge, Sweden

**Keywords:** (*R*)-[^11^C]PAQ, MMTV-PyMT mouse model of breast cancer, VEGFR-2, Paclitaxel, B20-4.1.1

## Abstract

**Background:**

The goal of the study was to assess the potential of the vascular endothelial growth factor receptor (VEGFR)-2-targeting carbon-11 labeled (*R*)-*N*-(4-bromo-2-fluorophenyl)-6-methoxy-7-((1-methyl-3-piperidinyl)methoxy)-4-quinazolineamine ((*R*)-[^11^C]PAQ) as a positron emission tomography (PET) imaging biomarker for evaluation of the efficacy of anticancer drugs in preclinical models.

**Methods:**

MMTV-PyMT mice were treated with vehicle alone (VEH), murine anti-VEGFA antibody (B20-4.1.1), and paclitaxel (PTX) in combination or as single agents. The treatment response was measured with (*R*)-[^11^C]PAQ PET as standardized uptake value (SUV)_mean_, SUV_max_ relative changes at the baseline (day 0) and follow-up (day 4) time points, and magnetic resonance imaging (MRI)-derived PyMT mammary tumor volume (TV) changes. Expression of Ki67, VEGFR-2, and CD31 in tumor tissue was determined by immunohistochemistry (IHC). Non-parametric statistical tests were used to evaluate the relation between (*R*)-[^11^C]PAQ radiotracer uptake and therapy response biomarkers.

**Results:**

The (*R*)-[^11^C]PAQ SUV_max_ in tumors was significantly reduced after 4 days in the B20-4.1.1/PTX combinational and B20-4.1.1 monotherapy groups (*p* < 0.0005 and *p* < 0.003, respectively). No significant change was observed in the PTX monotherapy group. There was a significant difference in the SUV_max_ change between the VEH group and B20-4.1.1/PTX combinational group, as well as between the VEH group and the B20-4.1.1 monotherapy group (*p* < 0.05). MRI revealed significant decreases in TV in the B20-4.1.1/PTX treatment group (*p* < 0.005*)* but not the other therapy groups. A positive trend was observed between the (*R*)-[^11^C]PAQ SUV_max_ change and TV reduction in the B20-4.1.1/PTX group. Statistical testing showed a significant difference in the blood vessel density between the B20-4.1.1/PTX combinational group and the VEH group (*p* < 0.05) but no significant difference in the Ki67 positive signal between treatment groups.

**Conclusions:**

The results of this study are promising. However, additional studies are necessary before (*R*)-[^11^C]PAQ can be approved as a predictive radiotracer for cancer therapy response.

## Background

Positron emission tomography (PET) is one of the fastest growing medical imaging modalities worldwide and, alone or in combination with other non-invasive imaging techniques, is used as a scientific and diagnostic tool in many medical fields [1].

A variety of radiopharmaceuticals has been established for imaging with PET, allowing visualization, monitoring, and measuring molecular and cellular events in the living organisms with high sensitivity (reviewed in [2]).

Angiogenesis is driven by potent pro-angiogenic factors and signaling molecules, including growth factors and growth factor receptors [3]. One of the most prominent of these is vascular endothelial growth factor (VEGF)A, also known as vascular permeability factor [4]. Although VEGFA binds with both VEGFR-1 and VEGFR-2 receptors, it is commonly agreed that VEGFR-2 is the key mediator of the mitogenic, angiogenic, and microvascular permeability-enhancing effects of VEGF [5]. The overexpression of VEGF/VEGFR-2 by tumor endothelium is associated with increased angiogenesis, metastatic spread of tumor cells, and with poor prognosis in cancer patients [6].

A review of the current literature reveals ten anti-VEGF/VEGFR-2 drugs, approved by the US Food and Drug Administration (FDA), in clinical use as monotherapy or in combination for the treatment of various types of cancer. These drugs include antibodies and their fragments (Fab-fragments, single chains), proteins, peptides, and tyrosine kinase inhibitors (TKIs) [7, 8]. Bevacizumab (BVZ, Avastin®), a humanized anti-VEGF monoclonal antibody, was the first anti-angiogenic drug approved by the FDA in 2004–2006 for the treatment, in combination with chemotherapy, of patients with metastatic colorectal cancer, advanced non-small cell lung cancer, and renal cell carcinoma (reviewed in [9]). The European Medicines Agency retains BVZ in combination with paclitaxel (PTX) or capecitabine as the first-line treatment of patients with HER-2-negative locally recurrent/metastatic breast cancer [10].

The types of drugs mentioned above as well as several other molecules have demonstrated potential as PET ligands for non-invasive in vivo imaging of the VEGF/VEGFRs. In preclinical studies, several zirconium-89 [11, 12] and copper-64 [13] labeled antibodies showed promising ability to visualize and quantify VEGF/VEGFR levels in tumor vasculature. In clinical applications, PET with zirconium-89-labeled BVZ has been used for assessment of anti-angiogenic treatment efficacy in patients with metastatic renal cell carcinoma [14] and non-small cell lung cancer patients [15].

Most TKIs are multi-targeting agents. There is therefore a growing interest in the discovery of TKIs with improved target selectivity, affinity in the subnanomolar range, and capability to penetrate the cell membrane [16]. A number of TKIs that block the adenosine triphosphate binding site of the VEGFRs TK domain and inhibit receptor-mediated intracellular signaling, thereby reducing angiogenesis, are among the new candidates. One of these, vandetanib (ZD6474) is an orally active VEGFR-2 TKI that has been shown to suppress tumor-induced angiogenesis in several xenograft models [17].

3-Piperidinylethoxy-anilinoquinazoline (PAQ) is an analog to vandetanib with 40 times stronger inhibitory properties for the VEGFR-2 [18]. The (*R*)-PAQ molecule has two stereoisomers, *S* and *R*, with IC_50_-values of 10 and 1 nM, respectively, for the VEGFR-2 at competitive concentrations of 2 μM adenosine triphosphate. Regarding the specific binding in comparison to others RTKs, the VEGFR-2 *R*-isomer had a 200-fold higher affinity versus epidermal growth factor receptor (EGFR) compare to only a 10-fold difference for *S*-isomer versus EGFR. This data convinced us to perform the further studies with the pure *R*-isomer.

We have previously described the synthesis and carbon-11 labeling of PAQ to yield (*R*)-[^11^C]PAQ [19] and demonstrated that the radiotracer uptake correlated with high VEGFR-2 expression in primary tumors and during metastasis development [20].

The current study aimed to examine the capability of using the (*R*)-[^11^C]PAQ VEGFR-2-targeting for monitoring anticancer treatment in the MMTV-PyMT/FVB (PyMT) transgenic mouse breast cancer model. This animal model was chosen for its translational capacity, i.e., developing adenocarcinomas with metastatic potential and its similarities to human luminal B breast tumors [21]. The PTX/BVZ therapies and the dosing selected for this study were based on our previous pilot studies and other preclinical studies [22, 23]. PTX is a mitotic inhibitor commonly used as a first-line chemotherapy. When combined with BVZ, progression-free survival and objective response rate in patients with metastatic breast cancer were significantly improved compared to PTX alone [24, 25]. Since BVZ has a high specificity for only human VEGFA, its murine analog B20-4.1.1 was used in the present study [26].

## Methods

### (*R*)*-*PAQ synthesis and radiolabeling

A detailed protocol of the (*R*)-PAQ precursor synthesis is described in [19] and the ^11^C- radiolabeling protocol in [20]. Briefly, (*R*)-PAQ was first synthesized and evaluated in vitro by Hennequin et al. [18]. The (*R*)-PAQ precursor, *N*-desmethyl, was synthesized with an enantiomeric purity of > 99%. (*R*)-[^11^C]PAQ was synthesized by the reaction of *N-*desmethyl-PAQ (1 mg) with [^11^C] methyl-iodide (CH_3_I), produced by standard (LiAlH_4_/HI) methods used at the Karolinska Hospital/Institutet, in a mixture of K_2_CO_3_ (10 mg) in dimethylformamide (0.4 ml). The (*R*)-[^11^C]PAQ radiochemical purity was > 98% as determined by radio analytical liquid chromatography. The decay-corrected radiochemical yield was approximately 10% at the end of synthesis. The synthesis time was 40–50 min. The (*R*)-[^11^C]PAQ identity was confirmed by co-elution with the unlabeled reference standard.

### Animal model and treatment groups

The PyMT transgenic mice were transferred from the breeding facility at the Wallenberg laboratory to Department of Comparative Medicine, Karolinska Institutet, Sweden, and were acclimated 1 week prior to the first PET and MRI sessions. The PyMT mice (female, 12–13 weeks old) included in the study (*n* = 12) underwent (*R*)-[^11^C]PAQ PET and MRI examinations before the treatment start (baseline, day 0). At this late stage of tumorigenesis, the PyMT mice develop tumors in all ten mammary glands. Three mammary glands, localized on each parasagittal section of the mouse body in the neck and thorax area and two mammary glands in abdomen/inguinal, developed tumors that merge to form four large tumor regions (Fig. 2a). Shortly after the baseline PET and MRI scans, mice (*n* = 3 mice, 11–12 tumors per group) were divided into the treatment groups and challenged with a single intraperitoneal dose of the VEH (control), PTX (10 mg/kg, Sigma), or BVZ murine analog mAb B20-4.1.1 (5 mg/kg) as monotherapies, or in combination. PTX stock solution was prepared in 50% Cremophor El (Sigma) and 50% ethanol, further diluted in saline (0.9% sodium chloride) to a final concentration 5% Cremophor El and 5% ethanol immediately prior to injection in the mice. Post-treatment PET and MRI examinations were performed 4 days after the single administration of the drugs (day 4).

### PET/MRI imaging and data analysis

#### PET imaging

Animals were anesthetized with isoflurane (5% initially and then 1.5% to maintain anesthesia) and placed on a heated pad (37 °C), with most of the body in the field of view (7.68 cm). The anesthetic concentration was regulated using an E-Z anesthesia vaporizer and blended with 7:3 air/O_2_ delivered through a Microflex non-rebreather mask (Euthanex Corporation, PA). (*R*)-[^11^C]PAQ (diluted in physiologically buffered saline to a final concentration of < 10% ethanol; max volume of 200 μl) was administered by a single injection via the tail vein. Doses of (*R*)-[^11^C]PAQ injected ranged from 2.6 to 7.4 MBq/g (specific activity typically of 1000–2000 GBq/μmol at injection). The list mode data were collected using the MicroPET Focus 120 scanner (CTI Concorde Microsystems) continuously over 60 min starting at the time of injection and reconstructed by standard 2D filtered back projection using a ramp filter. The matrix size of the reconstructed images was 256 × 256 × 95 with a spatial resolution 1.3 mm at the center of the field of view. The (*R*)-[^11^C]PAQ radiotracer uptake in mammary tumors is quantified as standard uptake values, SUV_max_ (the single maximum voxel value), and SUV_mean_ (the average SUV) within a volume of interest (VOI) [27]. The SUV values were calculated in relation to body weight from data summed from 30 to 60 min after intravenous administration of (*R*)-[^11^C]PAQ.

#### MR imaging

Immediately after PET scans, the animals were scanned with MRI within the same anesthesia session. The animal was positioned in the breathing mask of the MR-compatible rig. The placement of the animal was guided by the outline from the PET session. The temperature of the animal was controlled by warm air where the heating of the air was controlled by a feedback system to maintain the rectal temperature at 37 °C (SA-instruments, Stony Brook, USA). Respiration was monitored using a respiration pillow (SA-instruments, Stony Brook, USA) taped to the back of the animal. MRI data were acquired at 9.4T using a horizontal bore system (Varian, Yarnton, UK), equipped with a circularly polarized birdcage coil (an inner diameter of 72 mm) (Rapid Biomedical, Würzburg, Germany). The T1-weighted images were acquired using 3D gradient echo sequence (matrix size 512 × 192 × 256, field of view 100 × 45 × 64 mm^3^, time to echo 3.6 ms, flip angle 65° two averages, recovery time 7.6 ms). The VOIs were drawn over four mammary tumor regions of every mouse (two regions, left and right, covering the cervical and thoracic and two regions, left and right, covering the abdominal and inguinal mammary gland tumors, respectively). The percent change in tumor volume (TV) from baseline was calculated for each tumor region using the formula: [(TV day 0 − TV day 4)/TV day 0] × 100%.

The MRI images were first saved in the NIFTY format on the MR scanner, imported into Amide [28] and saved in the Siemens/Concorde format and imported in the Inveon Research Workplace software. Four VOIs were drawn manually in each MRI image and transferred to the corresponding PET images for quantification of radiotracer uptake. Immediately after the MR imaging on day 4, the mice were euthanized and the tumor tissue was removed for fixation in a 10% formalin solution.

### Histology/immunohistochemistry/image analysis/quantification

The tumor’s physiological characteristics, angiogenesis and cellular proliferation, were examined ex vivo by analyzing CD31 and Ki67 immunoreactivity, respectively. Routine histological analysis of hematoxylin and eosin (H&E)-stained tumor sections was performed on formalin-fixed paraffin-embedded tumors in order to investigate the variability of their phenotype. For immunohistochemistry (IHC), five sections were prepared from each tumor at 100 μm intervals, representing different tumor areas. The tumor sections (4 μm) were mounted on SuperFrost™ (Thermo Scientific)™ Plus object glasses, baked at 60 °C for 30 min. Antigen retrieval was performed for 20 min at 94 °C (pH 6.0). The sections were incubated with rabbit anti-Ki67 monoclonal (Cell Signaling Technology, #12202) and rabbit anti-CD31 polyclonal (Abcam, ab28364) primary antibodies. After several washes, the sections were incubated with biotinylated goat anti-rabbit (Dako) secondary antibodies and visualized with a 3, 3′-diaminobenzidine (DAB) solution (Dako).

For the Ki67 digital image analysis, slides were scanned with a 3DHistech Pannoramic Midi slide scanner, using a × 20 objective. Scans were inspected and analyzed using the 3D Histech Pannoramic viewer version 1.15.3. Manual annotations were made to exclude non-tumor tissues such as muscle and salivary gland that might interfere with the analysis. The annotated areas were then subjected to a tiered analysis using 3DHistech’s quant modules, histoquant, for identification and selection of viable tumor tissue, followed by nuclear quant for detection of positive nuclei within viable tumor area, thus excluding necrotic regions and cystic areas from the analysis. Because of unreliable detection of negative nuclei against tissue background, detected positive nuclei were then related to the area of viable tumor tissue. The Ki67 proliferation index of each tumor was calculated as a ratio between the number of Ki67 positive nuclei and the viable tumor area.

For tumor vascularization analysis, the three regions with the highest density of CD31-positive microvessels were selected within each tumor whole-slide image, using the 3D Histech Pannoramic viewer at the × 4 magnification. The quantitative data analysis was performed for each region (the area 0.41–0.45 mm^2^) within the tumor at the × 20 magnification, sufficient for the accurate visualization of microvessels. The microvessel density (MVD) was estimated for each tumor whole-slide image as the sum of CD31-positive vessels in all three regions.

### Statistics

Multiple independent populations were compared with the Kruskal-Wallis test followed by Dunn’s multiple comparison tests. Correlations were established by the Spearman rank correlation test. Wilcoxon signed-rank test was used to test whether variables such as SUV_mean_, SUV_max_, and TV changed from baseline to day 4 in each treatment group. All tests were conducted in R version 3.3.3 or GraphPad Instat, version 3.04 (GraphPad Software Inc.). Graphs were produced in GraphPad Prism, version 5.03. *p* values of 0.05 or less were considered significant.

## Results

### Evaluation of treatment efficacy in PyMT mice using (*R*)-[^11^C]

#### PAQ PET and MR imaging

First, the PyMT mice underwent PET imaging for examination of baseline (*R*)-[^11^C]PAQ radiotracer uptake, followed up by MRI scans in order to determine the size of mammary neoplasms prior to treatment. From our previous experience, defining the tumor border solely on the basis of the variable target expression/tracer uptake in (*R*)-[^11^C]PAQ PET is difficult, and therefore, the superior soft tissue resolution capability of MRI was used to determine the VOIs.

**Fig. 1:**
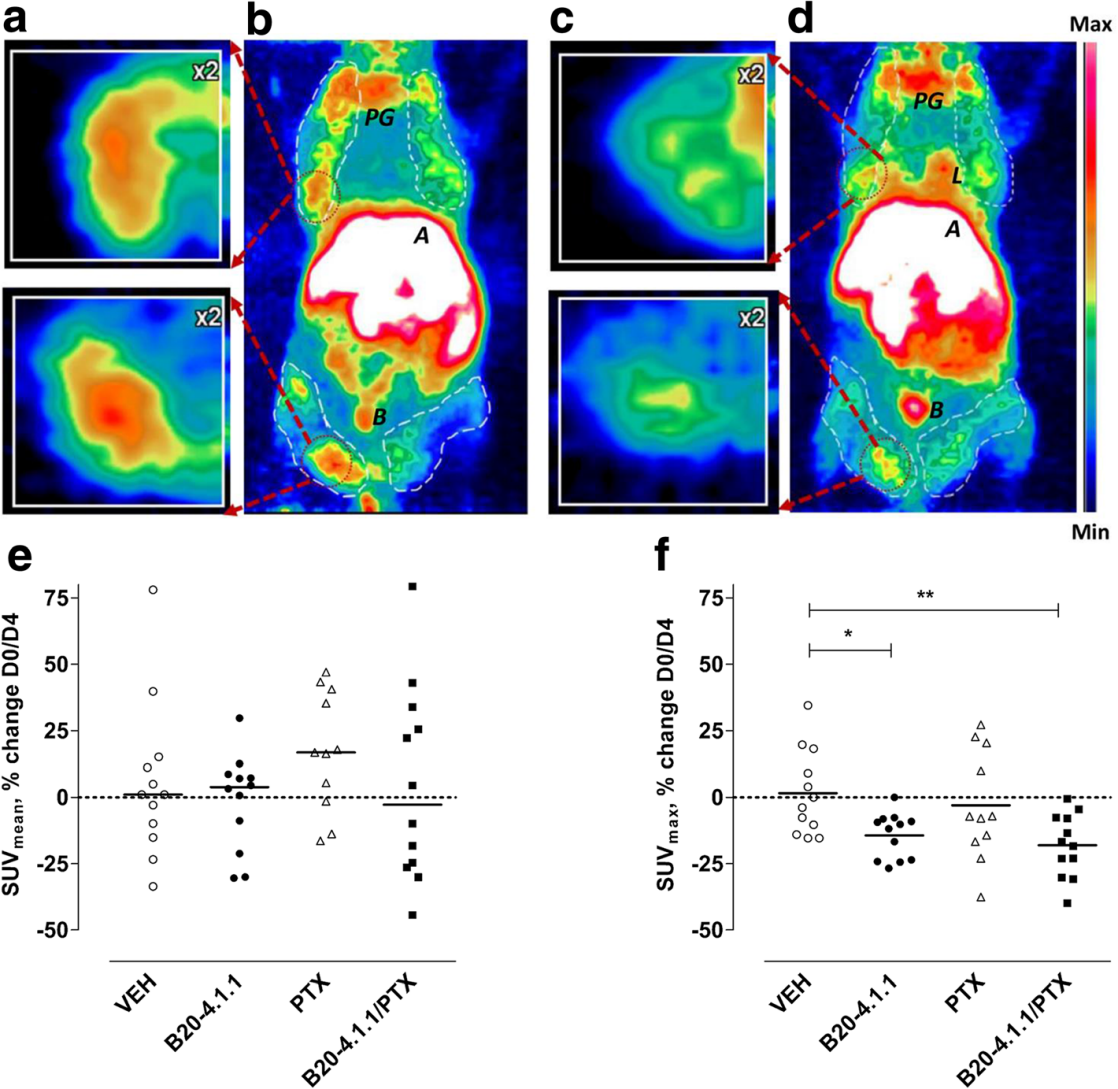
Therapy response assessments in PyMT mice with (*R*)-[^11^C]aPAQ PET imaging. The (*R*)-[^11^C]PAQ uptake in PyMT mouse **a**, **b** before (baseline, day 0) and **c**, **d** 4 days after B20-4.1.1/PTX combinational treatment (shown as example). The twofold magnified boxed areas (**a**, **c** upper and lower images) show transverse plane through the corresponding mammary tumors area (red dotted circles). The white dashed areas on **b** and **d** represent the mammary tumor areas, included in PET/MRI analyses. PG, parotid glands; A, abdomen; B, bladder. All images are scaled to the same color scale. Quantification summary of (*R*)-[^11^C]PAQ uptake **e** SUV_mean_ and **f** SUV_max_ values as a percentage of change from baseline, day 0 to day 4 in all treatment groups (*n* = 11–12 tumors/group). The comparisons between the groups are shown (Kruskal-Wallis test, *p < 0.05 and **p < 0.01). Each individual tumor is presented as a dot. The horizontal lines indicate the median values

To characterize the angiogenic status of the heterogeneous mammary tumors, the (*R*)-[^11^C]PAQ radioactivity SUV_mean_ and SUV_max_ values were acquired for 47 tumors, divided among the four treatment groups. Table 1 shows the median values for tumor volumes, SUV_mean_, and SUV_max_, estimated for every group at baseline (day 0) and at the post-treatment time (day 4). The (*R*)-[^11^C]PAQ accumulation provided good visualization of the tumors’ angiogenic areas within 60 min after radiotracer administration (Fig. 1a–d).

**Table 1:** The tumor volumes (TV) and (*R*)-[^11^C]PAQ SUV values at baseline (day 0) and at treatment follow-up (day 4)

Treatment (# analyzed tumors)	TV(mm^3^) Median ± SD Day 0	TV(mm^3^) Median ± SD Day 4	SUV_mean_ Median ± SD Day 0	SUV_mean_ Median ± SD Day 4	SUV_max_ Median ± SD Day 0	SUV_max_ Median ± SD Day 4
VH (*n* = 12)	780 ± 320	920 ± 415	0.37 ± 0.09	0.41 ± 0.08	1.1 ± 0.18	1.1 ± 0.15
B20-4.1.1 (*n* = 12)	920 ± 320	735 ± 420	0.43 ± 0.07	0.42 ± 0.06	1.2 ± 0.16	0.91 ± 0.14
PTX (*n* = 11)	873 ± 475	868 ± 615	0.36 ± 0.07	0.46 ± 0.06	1.0 ± 0.18	1.1 ± 0.15
B20-4.1.1/PTX (*n* = 12)	760 ± 335	525 ± 370	0.36 ± 0.23	0.39 ± 0.1	1.1 ± 0.18	0.88 ± 0.11

*SD* standard deviation

The PET data, calculated as each individual tumor’s percent change from baseline to day 4, were assessed for statistical significance within each treatment group and between the treatment groups (Fig. 1e, f). Comparing the SUV_mean_ values, there was no significant change from baseline to day 4 (*p* > 0.05), tested in each treatment group separately (Fig. 1e). Furthermore, the change from baseline to day 4 was not significantly different between the treatment groups or the VEH group (*p* > 0.05) (Fig. 1e).

Comparing the SUV_max_ values within a group, there was a significant change from baseline to day 4 in the B20-4.1.1/PTX combination group (*p* < 0.0005) and B20-4.1.1 monotherapy treatment group (*p* < 0.003), but no significant change in either PTX monotherapy group (*p* > 0.5) or VEH group (*p* > 0.5) (Fig. 1f). Furthermore, when comparing the change in SUV_max_ between the groups, the test showed significant difference in SUV_max_ change between VEH group and B20-4.1.1/PTX combination treatment group (*p* < 0.005), as well as VEH and B20-4.1.1 (*p* < 0.05) but not between VEH and PTX monotherapy groups (Fig. 1f).

**Fig. 2:**
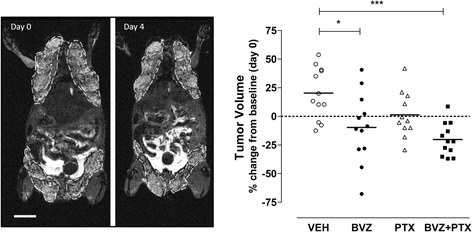
Therapy response assessments in PyMT mice with MR imaging. **a** Representative T1-weighted coronal MR images of the PyMT mouse, treated with B20-4.1.1/PTX combination (shown baseline, day 0 and 4 days after the treatment, day 4). Tumor ROIs are outlined with white dashes. Scale bar 10 mm. **b** Quantification summary of the therapeutic response to treatment (*n* = 11–12 tumors/group). The comparisons between the groups are shown (Kruskal-Wallis test, *p < 0.05 and ***p < 0.001). The horizontal lines indicate the median values

We used MRI-derived TV data to quantify the differences in response within and between the treatment groups and correlated it with radiotracer uptake during the treatment period. As an example, Fig. 2a shows representative T1-weighted coronal MRI images of the PyMT mouse from B20-4.1.1/PTX treatment group before (left image) and 4 days after the treatment start (right image). The TV was significantly reduced in the B20-4.1.1/PTX combination therapy group (*p* < 0.005) but not in B20-4.1.1 or PTX monotherapy groups (*p* > 0.05). The TV change was significantly different between the VEH (control) group and B20-4.1.1/PTX combination treatment group (*p* < 0.05), as well as B20-4.1.1monotherapy group (*p* < 0.05) but not the PTX monotherapy group (Fig. 2b).

**Fig. 3:**
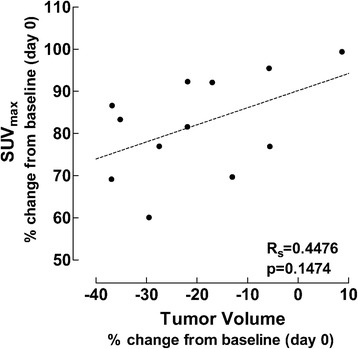
Scatter plot showing the relationship between the (*R*)-[^11^C]PAQ SUV_max_ values and the MRI-derived mammary tumor volume changes (percentage of change to baseline) in the B20-4.1.1/PTX treatment group only. Spearman’s rho = 0.45, *p* > 0.05

Graphical illustration of the (*R*)-[^11^C]PAQ SUV_max_ changes compared with mammary TV reduction suggested a positive correlation in the B20-4.1.1/PTX group (Fig. 3). However, Spearman’s rank correlation test did not show a significant correlation in any of the treatment groups nor in the VEH group (*r*_s_ − 0.45, *p* > 0.05).

### Evaluation of treatment efficacy in PyMT mice by Ki67 and CD31 immunohistochemistry

Histopathological evaluation of 12 end-stage tumors (three representatives per each group) by H&E staining confirmed their diverse histological patterns and intra-tumoral heterogeneity. 7/12 tumors were classified as predominantly solid adenocarcinomas, 1/12 as multi-lobular pattern with multiple smaller solid lobules, and 4/12 tumors as variable phenotype. All tumors had necrotic areas, commonly in the central tumor regions and the mitotic rate, as defined within viable tumor tissue at the × 40 magnified microscopic view, varied from 5 up to 10.

**Fig. 4:**
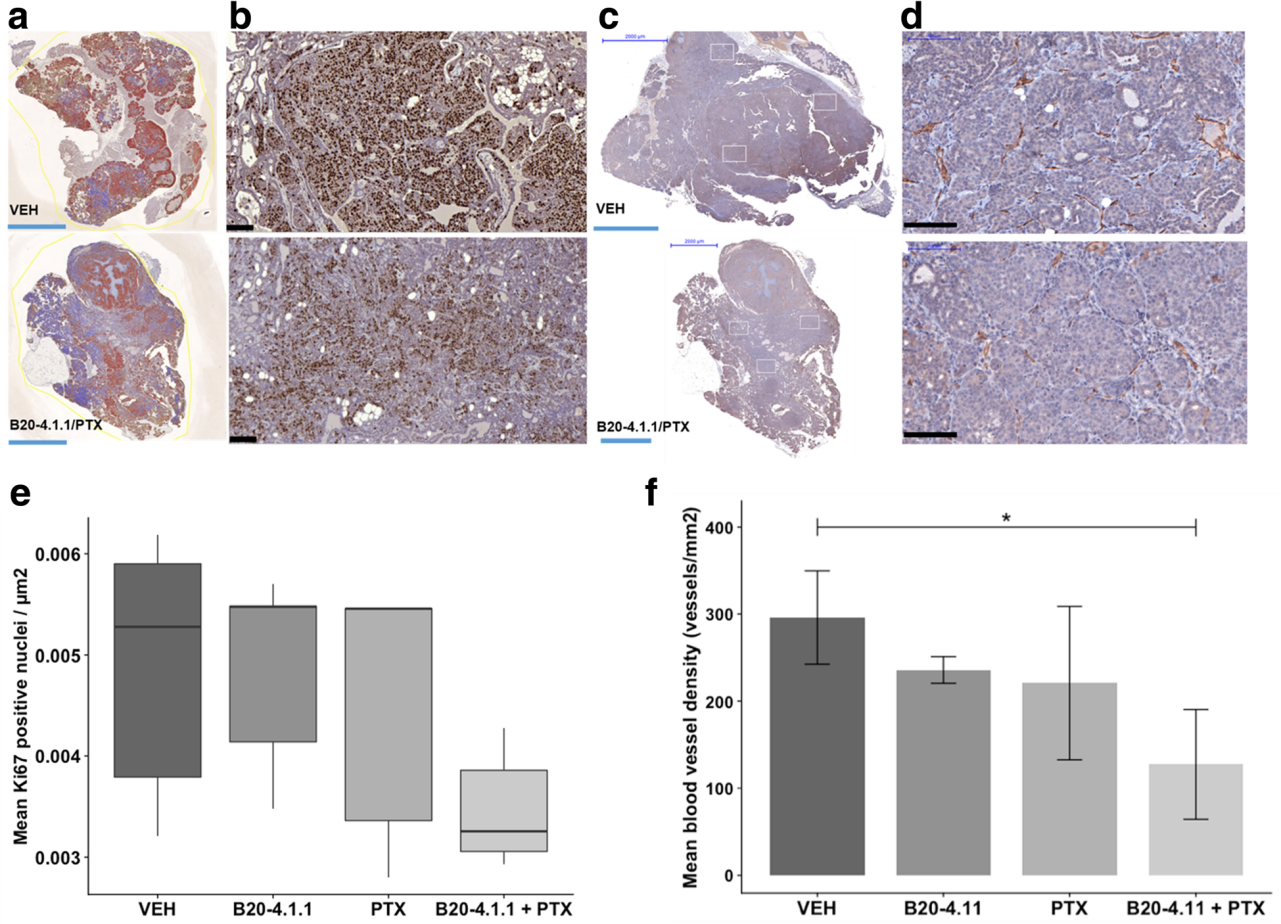
Evaluation of treatment efficacy on PyMT mammary tumors’ proliferation and angiogenesis. Immunostaining of **a**, **b** Ki67 and **c**, **d** CD31 expression in PyMT mammary tumors, treated with B20-4.1.1/PTX combination. The whole tumor cross-sections (**a**, **c**) and representative magnified tumor areas (**b**, **d**) shown for VEH (upper panel) and B20-4.1.1/PTX combinational treatment (lower panel). Ki67-positive signals within the tumors are shown in brown. The boxed areas indicate tumors “hotspots” with the highest density of microvessels, selected for quantification. Scale bars: blue = 20 mm; black = 100 μm. **e** Quantitative assessment of mean Ki67-positive signal per micrometer of tumor viable area in each sample (*n* = 5 in each treatment group). The Ki67 proliferation index was considerably low in B20-4.1.1/PTX combination group (the mean value, 0.0035) in comparison to the VEH (0.0049), B20-4.1.1 (0.0048), or PTX (0.0045) monotherapy groups; however, no statistically significant difference was observed (Kruskal-Wallis test, *p* > 0.05). A box plot displays the range of variation of the Ki67 proliferation index for each treatment group (the horizontal lines indicate the median value). **f** Tumor vessel density, in vessels/μm^2^. A significant difference was found between the VEH and B20-4.1.1/PTX groups (the mean values 296.2 and 127.2 vessels/μm^2^, respectively), but not between the VEH and B20-4.1.1 or PTX groups (the mean values 236 and 220.7 vessels/μm^2^, respectively; Dunn’s multiple comparisons test, *p < 0.05). Error bars represent standard deviations

We defined tumor viable areas (necrotic and cystic regions were excluded) and quantified the Ki67 positive nuclei in the tumor cross-sections. The highest number of Ki67 positive nuclei was observed in VEH (control)-treated tumors (Fig. 4a, b, upper panel). It is apparent from Fig. 4a, b, lower panel, that the Ki67 proliferation index was considerably lower in the B20-4.1.1/PTX combination group compared to other treatment groups; however, the Kruskal-Wallis test did not show any statistically significant differences between the treatment groups (*p* > 0.05) (Fig. 4e). Furthermore, we analyzed the mammary tumor vascularization in VEH-treated tumors (Fig. 4c, d, upper panel) and B20-4.1.1/PTX combination group (Fig. 4c, d, lower panel). We observed a significant difference in tumor microvascular density only in the B20-4.1.1/PTX treatment group compared to other groups (*p* < 0.05*)* (Fig. 4f).

## Discussion

This study investigated the potential capability of using the VEGFR-2-targeting (*R*)-[^11^C]PAQ PET radiotracer to monitor and evaluate the efficacy of anticancer treatment in the PyMT mouse model of breast cancer. The study was performed in female mice at the late stage of malignancy, which is characterized by high expression levels of VEGFR-2, CD31, and other proangiogenic factors in the mammary tumor vasculature [29]. Histological profiling of the PyMT tumors, also included in this study, showed typically heterogeneous histology patterns, with irregularly distributed necrosis and more clustered areas with higher mitotic index and angiogenic activity. These factors also contribute to the heterogeneous distribution of the VEGFR-2-targeting (*R*)-[^11^C]PAQ radiotracer within the tumors observed here.

The quantitative analysis of the (*R*)-[^11^C]PAQ PET data revealed a significant reduction of the radiotracer uptake (SUV_max_) in the PyMT mammary tumors within both the B20-4.1.1/PTX combination and B20-4.1.1 monotherapy treatment groups compared to the control (VEH group). The result was statistically significant in these two groups when analyzed both within the group and in comparison to the control (VEH group).

Significant reductions of the mammary TVs during therapy, as measured with MR imaging, were observed only within the B20-4.1.1/PTX combination treatment group, and only the modest effects were observed in mice treated with PTX and B20-4.1.1 monotherapies. It is important to note that, even though TV was not significantly reduced in the B20-4.1.1 group, the TV reduction was higher in this group than in the VEH group. We detected a large variation in relative TV changes within the B20-4.1.1 group, even though there was a consistent reduction in the SUV_max_ from day 0 to day 4. We interpret this as an effect of anti-angiogenic treatment with B20-4.1.1 on the tumor microvasculature leading to a decreased uptake of the targeting radiotracer, but not to a reduction in tumor size. Although it appears that PTX, which is not as closely associated with angiogenesis, does not lead to a systematic reduction in SUVmax (compared to VEH), a corresponding lack of effect of PTX on tumor volume or number of Ki67 positive cells prevents us from presenting this as evidence of tracer selectivity for tumor angiogenic sites.

We observed a trend toward a positive correlation between the (*R*)-[^11^C]PAQ SUV_max_ changes and the mammary TV reductions in the B20-4.1.1/PTX group. The moderate (*r*_s_ = 0.45) correlation could possibly be due to the large spread in initial tumor sizes (and therefore their baseline characteristics) in the treatment group. In this model and study protocol, the (*R*)-[^11^C]PAQ SUV_max_ appeared to be a more sensitive to treatment than SUV_mean_. The SUV_mean_ values were found to be more variable due to high diversity in tumors histological pattern and intra-tumoral heterogeneity.

The current study showed that the microvascular density of the PyMT tumors was significantly lower in the B20-4.1.1/PTX combination treatment group than in the other groups. A number of preclinical studies have demonstrated that anti-angiogenic drugs enhance chemotherapy delivery and penetration, improving tumor response by remodeling the vasculature [30, 31]. Dickson et al. showed that a single dose of the anti-VEGF antibody BVZ caused an overall decrease in tumor microvascular density by destroying the immature vessels and improving tumor perfusion and responsiveness to chemotherapy in neuroblastoma xenografts [32].

PAQ acts as a competitive inhibitor of the ATP-binding pocket at the catalytic intracellular tyrosine-kinase (TK) domain of VEGFR-2. Activation of VEGF-2 by VEGF results in the formation of receptor dimers, following by cross-phosphorylation of the intracellular TK domains of the receptors and intracellular signal transduction [33].

PAQ binds to the TK domain only when the receptor is in its inactive conformation, at which time the ATP pocket is available (i.e., in the absence of ligand binding/dimerization/phosphorylation). Thus, the balance between all the factors that affect the availability of the ATP-binding domain at a given time will determine the amounts of radiolabeled PAQ retained at that imaging session. The production and release of VEGF are higher when the tumors are fast growing and hypoxic [34], as in the PyMT model. The higher the levels of VEGF, the greater the probability that it will interact with the receptor and the lower the number of “free” ATP-binding sites. Activation by VEGF results in receptor internalization, endocytosis, and recycling, but the VEGFR-2 undergoes constitutive endosome-to-plasma membrane recycling even in the absence of ligand [35]. The dynamics of this recycling will affect the speed at which the ATP-binding sites once again become available for PAQ binding. VEGF-targeted therapies like BVZ would initially lead to a decrease in the VEGF available for binding with the receptor. Therefore, in the acute phase, the relative availability of the ATP-binding sites for (*R*)-[^11^C]PAQ could increase. However, with time, the tumor endothelial cells die and the blood vessel regression is achieved (reviewed in [36]), which would lead to a decreased retention of (*R*)-[^11^C]PAQ. Dynamic changes in the concentration of circulating VEGF and the contribution of host stromal VEGF make it difficult to estimate the amount of antibodies for efficient blocking [26].

B20-4.1.1 is a cross-species monoclonal antibody targeting both human and murine VEGF [26], and it has been used to treat various preclinical tumor models [37, 38]. Anti-VEGF blocking depends on both the tumor context and treatment. Bagri et al. [39] have evaluated the effects of anti-VEGF treatment in a diverse panel of tumor xenografts and genetic mouse models of cancer. Their studies concluded that continuous VEGF suppression with B20-4.1.1 provided additional benefit in reducing tumor growth when combined with chemotherapy. However, there have been only a few reports on the use of B20-4.1.1 in the MMTV-PyMT model. A recently published study [40] demonstrated that long-term monotherapy with B20-4.1.1 caused significant tumor growth inhibition in the PyMT model and affected microvessel density in a similar manner as the two anti-angiogenic TKIs, nintedanib, and dovitinib.

In contrast to B20-4.1.1 monotherapy and B20-4.1.1/PTX combination therapy, no significant treatment-induced changes in TV and radiotracer uptake were observed in the PTX monotherapy group. PTX stabilizes the microtubules in proliferating cells by blocking them from the progression of mitosis, and it induces apoptosis in cancer cells [41, 42]. Recent studies have, however, demonstrated that PTX induced resistance to chemotherapy and promoted pulmonary and lymphatic metastasis in the PyMT model. Volk-Drapper et al. [43] have shown that repeated PTX treatment caused pro-oncogenic and intratumoral inflammatory changes in the PyMT mammary tumors through activation of the Toll-like receptor (TLR4). Another study [44] showed that high-dose PTX treatment in PyMT mice caused increased macrophage infiltration that protected tumors from cell death and facilitated tumor progression and metastasis.

In our study, possible PTX effects on proliferation were examined by immunohistochemical determination of the proliferation marker Ki67. Ki67 has been identified as an independent prognostic factor in breast cancer patients [45] and has also been used to evaluate PyMT tumor proliferating activity in preclinical studies [46]. In our study, a single dose of PTX did not alter the fraction of Ki67 positive cells in any treatment group. Only the tumors treated with combined B20-4.1.1/PTX showed clearly reduced Ki67 proliferation index on day 4 compared to other groups, though this difference did not reach statistical significance.

The structural analogs of (*R*)-[^11^C]PAQ, carbon-11-labeled vandetanib, and chloro-vandetanib have been successfully developed for potential applications as VEGFR-2 radiotracers; however, they have yet to be evaluated in vivo [47]. In this study, we used the PAQ synthesis protocols that we have used in previous validations of this radiotracer [18]. General radiolabeling and purification procedures with carbon-11 by alkylation reactions with [^11^C]methyl iodide are well-established methods. The encouraging results obtained during the [^11^C]PAQ evaluation in vitro and in vivo motivated us to here further evaluate the (*R*)-[^11^C]PAQ in additional disease models. However, fluorine-18 is an attractive PET radioisotope due to its longer half-life (permitting, for example, multiple studies from the same batch and higher imaging resolution), and future comparative studies with (*R*)-[^18^F]PAQ could be of interest. Prabhakaran et al. [48] have developed and synthesized the fluorine-18-labeled fluoroethyl analog of (*R*)-[^11^C]PAQ, (*R*)-[^18^F]FEPAQ. The authors have demonstrated tracer’s specific selectivity for VEGFR-2 in human glioblastoma frozen sections, though the tracer has not yet, to our knowledge, been evaluated in vivo.

In the clinical setting, BVZ combined with paclitaxel failed to show an overall survival benefit in metastatic breast cancer patients [49]. Several mechanisms of resistance to VEGF-targeted therapy have been suggested; among them are a complex interaction between tumor cells and stroma, an increased aggressiveness of the tumor caused by hypoxia (and thus new mutations), hypoxia-induced increase of cancer stem cells, and an activation of alternative pro-angiogenic signaling pathways [50]. Many other biomarkers for monitoring anti-angiogenic therapy have been studied, including circulating levels of pro-angiogenic factors, mutations in angiogenesis-related genes, tumor microvascular density, levels of vascular perfusion, hypertension, and in situ markers in tumor tissue [51, 52]. In addition, novel molecular and functional imaging probes targeted angiogenesis have been intensively developed and evaluated (reviewed in [53]). Despite the encouraging results with some of the above, there is still a lack of biomarkers that can be used to select a population of patients that would benefit from anti-angiogenic therapy.

In our study, we were able to demonstrate the promising capability of (*R*)-[^11^C]PAQ PET imaging for visualizing/quantifying treatment response. However, the single dose/short-term treatment was insufficient to produce statistically convincing evidence in the PyMT model. Future studies should examine the ability of (*R*)-[^11^C]PAQ to monitor therapeutic response in other dosing protocols. Similarly, multiple sequential (*R*)-[^11^C]PAQ PET studies over time could be attempted to see if an even more appropriate time than day 4 for the therapeutic read-out can be found.

## Conclusions

We have performed the first preclinical evaluation of the ability of (*R*)-[^11^C]PAQ PET imaging to determine anti-cancer treatment efficacy in PyMT transgenic mice. Although promising results were obtained, statistically significant correlations between radiotracer uptake and treatment efficacy biomarkers were not found. Additional preclinical studies are therefore necessary to further determine the predictive value of (*R*)-[^11^C]PAQ PET for evaluating therapeutic response.

## Abbreviations

(*R*)- [^11^C]PAQ: Carbon-11 labeled (*R*)-N-(4-bromo-2-fluorophenyl)-6-methoxy-7-((1-methyl-3-piperidinyl)methoxy)-4-quinazolineamine
MRI: Magnetic resonance imaging
PET: Positron emission tomography
PTX: Paclitaxel
SUV: Standardized uptake value
VEGFR-2: Vascular endothelial growth factor receptor
